# Salivary stimulatory effect of novel low level transcutaneous electro neurostimulator in geriatric patients with xerostomia

**DOI:** 10.1186/s12903-023-03049-0

**Published:** 2023-05-28

**Authors:** Ramya Ramadoss, Rajkumar Krishnan, Swarnalakshmi Raman, Rajashree Padmanaban, Nagarathinam Anbuelangovan, Rajalakshmanan Eswaramoorthy

**Affiliations:** 1 Department of Oral Biology, Saveetha Dental College, Chennai, India; 2 Department of Oral Pathology, SRM Dental College, Chennai, India; 3 Department of Stomatognathic Function and Occlusal Reconstruction, Graduate School of Biomedical Sciences, Tokushima University, Tokushima, Japan; 4 Centre of Advanced Study in Crystallography & Biophysics, University of Madras, Chennai, India; 5 Department of Biomaterials, Centre of Molecular Medicine and Diagnostics (COMMAND), Saveetha Dental College and Hospitals, Saveetha Institute of Medical and Technical Sciences (SIMATS), Chennai, India; 6 Department of Applied Chemistry, School of Applied Natural Science, Adama Science and Technology University (ASTU), PO. 1888, Adama, Ethiopia

**Keywords:** Xerostomia, Geriatric patients, Saliva, TENS, Unstimulated saliva

## Abstract

**Background:**

Xerostomia (dryness of the mouth) is one of the most common long-term consequences of ageing, and it causes a tremendous impact on the function and morphology of the salivary ductal system. As a consequence, it leads to a decrease in the amount of salivary output and also affects the overall quality of life. The purpose of this study was to determine whether electrostimulation using a custom designed transcutaneous electrical nerve stimulation (TENS) device will help to improve the quality of secreted saliva following electrostimulation.

**Methods:**

One hundred thirty-five participants underwent the intervention for three months, twice daily (80 Hz). Pre-intervention and post-intervention unstimulated saliva were collected. Parameters such as salivary pH, cortisol level, salivary antioxidants, total protein, the viscosity of saliva, and microbial carriage were analysed.

**Results:**

Salivary pH, cortisol, microbial cultures, viscosity, and antioxidants showed a significant difference at the end of the 3rd month (*p* < 0.05). Irrespective of the patient's age, gender, and common underlying systemic illnesses (diabetes and hypertension), a significant change in the quality of the salivary analytes was observed.

**Conclusion:**

The study emphasises the use of a custom designed TENS device in improving the quality of secreted saliva among old patients with oral dryness.

## Introduction

Xerostomia, i.e., dry mouth, is one of the most common long-term consequences of ageing, primarily due to morphological and functional effects on the salivary secretory and ductal systems that eventually result in decreased salivary output. Dry mouth can be caused by various etiological factors like age, local factors, systemic diseases, medications, etc. Prolonged dry mouth among older individuals significantly contributes to the negative impact on quality of life [1–6]. Usually, oral dryness is felt when there is a 50% decrease in salivary flow in any individual [7]. The subjective sensation of dry mouth with or without decreased salivary flow is referred to as "xerostomia." In this study, oral dryness was defined based on a reduced salivary output of ≤ 0.2 ml/min [7]. Although several pharmacological and non-pharmacological interventions are available to improve salivary output, solutions for long-term improvement in the quantity and quality of salivary output are insufficient. Certain pharmacological interventions help only with a transient improvement in salivary output, but symptoms may recur. Validated non-pharmacological interventions may help either as a primary intervention and/or neoadjuvant treatment option in improving the symptoms related to xerostomia [8].

Transcutaneous electrical nerve stimulation (TENS) is a device that uses electric current adjusted for pulse, frequency, and intensity for therapeutic purposes. It is known to be an effective, non-invasive, cost-effective, and self-administrable technique to relieve pain [9]. The technique is already used in several conditions, like endometriosis, arthritis, sports injuries, multiple sclerosis, fibromyalgia, painful diabetic neuropathy, spinal cord injury [10–12], and dental associated pain [13–15]. In the head and neck region, it is used in the management of temporomandibular joint dysfunction related to orofacial pain [13–15]. Few clinical trials have attempted to assess the effect of TENS on improving salivary flow by using electrical stimulation of the salivary glands. Normally, stimulators such as taste, smell, mastication, thought, etc. send the afferent signals/impulses via the glossopharyngeal nerve, facial nerve, and trigeminal nerve to the higher control centres in the medulla oblongata, which in turn directs signals to the efferent part of the reflex, leading to salivation. Studies that analysed the impact of TENS devices in improving salivary hypofunction postulated that the TENS device placed over the parotid gland is hypothesised to stimulate the postganglionic efferent fibres of the auriculotemporal nerve that improve the secretomotor drive of the parotid gland [16, 17]. Although mild to moderate improvement in saliva flow has been reported following the use of a TENS device, it is currently not applicable as a mainstream or complementary treatment as the therapy has not yet been validated [18].

A novel single-unit wireless salivary neuro electro stimulation device was designed to assess the effectiveness of improving the salivary flow among patients aged above 60 years presenting with oral dryness. The device was designed to generate a low-pulse electric current through miniature electrodes precisely positioned over the skin in the parotid region. Unlike the bulky, heavy TENS devices available on the market, the device used was compact and easier to use. The device showed promising results during the pilot run in improving the salivary output, compelling the need for further analysis among a larger population and to assess the quality of the salivary output [19]. This study aimed at a progressive evaluation of qualitative aspects of post-interventional whole unstimulated saliva secreted following the use of the novel single-unit wireless TENS device in patients aged > 60 years presenting with oral dryness Our null hypothesis is that the novel TENS device has no effect on the qualitative parameters of post-intervention, whole unstimulated saliva. The quantitative measure (i.e., salivary flow rate) of the post-intervention saliva has been published elsewhere but is mentioned here for emphasising the relevance between the salivary flow and its relevant functional measures that signifies the quality of the secreted saliva [19]. A thorough literature search revealed that the qualitative and quantitative assessment of both pre-interventional and post-interventional whole unstimulated saliva in patients above 60 years with mouth dryness is unique and the first of its kind.

## Materials and methods

This manuscript follows the STROBE Statement guidelines. The study design and protocol were approved by the Institutional Review Board (IRB) (REF SRMDC/ IRB/2015/Faculty/No.711).

### Study design, participants, and data sources

The study was conducted from June 2018 to March 2019. Participant recruitment for the study was based on age, a complaint of oral dryness, and reduced salivary flow. Clinically, oral dryness relates to decreased unstimulated salivary flow and mucosal wetness. Participants were screened for oral dryness using the "Challacombe Scale of Clinical Oral Dryness," a scoring tool that is easy to use and helpful in identifying and quantifying the severity of dry mouth based on key features of dry mouth. The Challacombe Scale of Clinical Oral Dryness is considered ideal for measuring dry mouth due to its comprehensive assessment approach, validated and reliable nature, clinical relevance, simplicity, and flexibility in different settings and populations. Compared to other scoring systems, the scale includes a range of oral dryness symptoms and their severity levels, making it useful for both diagnosis and treatment monitoring in patients with dry mouth. The scale also provides an overall and comprehensive approach to Clinical Oral Dryness as the scale measures subjective symptoms reported by the patient as well as objective clinical findings. Additionally, the Challacombe Scale has been widely adopted by healthcare professionals and researchers worldwide, indicating its usefulness and practicality [20, 21]. Patients whose age was 60 and above and who presented with a complaint of oral dryness and decreased salivary flow less than or equal to 0.2 ml/min were defined as criteria for recruitment into the study [6, 7]. Participants with common old age-related morbidities, including diabetes and hypertension, were also included in this study. However, participants with a known history of salivary gland pathologies like Sjogren's syndrome and rheumatoid arthritis were excluded. Participants who have undergone any surgical or radiotherapy procedure involving the salivary glands or those who are taking medications known to cause dry mouth [inhalatory glucocorticoids, opioids, benzodiazepines, diuretics, etc.] were also excluded from recruitment into the group. Because the study requires the use of low frequency pulse currents, participants with pacemakers, defibrillators, hearing aids, or cochlear implants were also excluded from the study.

### Collection of saliva

Participants were asked to use the device twice daily (morning and evening) for 10 min at a set frequency of 80 Hz. Pre and post-interventional saliva was collected using the "spit method" between 9 a.m. and 11 a.m. to standardise the effect of circadian rhythm on assessing the salivary parameters. Samples were collected using the spit method by asking participants to sit up and then spit the saliva into the tube for 10 min. All recruited participants were informed to refrain from habits like chewing gum or candy, eating, drinking any liquid or alcohol, or smoking for about an hour prior to the sample collection. The duration of 10 min for salivary collection was applicable for assessing the salivary flow rate but not for the measurement of several other variables; participants were asked to spit the saliva until a minimum of 5 ml was collected. Further, the collected saliva for analyte measurement was stored in a deep freeze refrigerator (-80 °C) for further analysis. In this study, "pre-interventional unstimulated saliva" refers to the saliva collected before electro-stimulation, and "post-interventional unstimulated saliva" refers to the saliva collected after the application of electro-stimulation. A summary of the methods for assessment of the salivary parameters assessed is listed in Table 1.2.3. Salivary Variables and Methodology (also refer to Table 1).

**Table 1:** Salivary parameters [21–33]

Variable	Principle	Method of assessment	Significance in assessment
1. Salivary pH	Measure Hydrogen Ion Concentration	Eutech pH meter, PC 2700 with ion sensitive probes (Thermo Fisher Scientific, Waltham, Massachusetts, USA)	Maintenance of balance in salivary pH (either alkaline or acidic) helps in balancing essential minerals, electrolytes, and enzymes that function to strengthen mineralized tooth structures and provide anti-inflammatory and immune function against plaque and microbial build-up. chiefly aiding in the digestive process
2. Salivary Cortisol	Measures the amount of free cortisol in saliva	Human Cortisol Elisa Kit (Bioassay Technology Laboratory, Shanghai, China)	Indicator of stress and an indirect measure of the quality of life of patients. Cortisol is a reliable indicator of stress, which is shown both in serum and saliva. Salivary cortisol is an accurate reflection of the cortisol in the blood and has proven to be reliable and valid. Few authors suggest salivary cortisol levels are more reliable as they measure unbound cortisol when compared to serum cortisol. Salivary cortisol levels may be influenced by many systemic and pathological factors. It is observed that salivary cortisol levels may vary based on the circadian rhythm. The baseline levels of the HPA axis show a well documented diurnal rhythm for cortisol secretion among individuals; it peaks within 60 min after waking and shows a gradual decline after reaching a nadir by around midnight
3. Salivary Antioxidant Capacity	Measures the antioxidant status of and evaluates the antioxidant response against the free radicals	FRAP assay using the ISA (Bioassay Technology Laboratory) kit	Measures the oxidative stress in the aging process and helps to assess the antioxidant defense status
4. Salivary Total Protein	Measures the quantity of total proteins produced in saliva	The BCA (bicinchoninic acid) assay was done using the colorimetric method (Elabscience kit, USA)	Salivary Total protein is a non-specific measure of the total amount of all proteins present in saliva; analytes may vary based on the physiological or diseased state and can vary in response to altered salivary flow. helps to assess the qualitative nature of saliva
5. Salivary Microbial Carriage	Assess the bacterial colonies present	*Streptococcus mutans and Lactobacillus colonies* count at a critical concentration of 105 CFU/mL	Salivary microbiota reflect the oral health and systemic health of the individual
6. Viscosity of Saliva	To assess the viscosity of saliva	analyzes it using Ostwald’s viscometer	Salivary viscosity relates to the salivary glycoprotein content contributing to the viscoelastic properties such as lubrication and humidification; aiding in mucosal integrity

The following salivary qualitative parameters were analysed from the collected whole saliva, both pre-interventional (Baseline) and post-interventional (12th week) samples: pH, viscosity, cortisol, total protein, FRAP (Ferric reducing antioxidant power) assay, and microbial carriage *(streptococcus mutans and lactobacillus count)*.

1. **Salivary Flow Rate:** Pre- and post-interventional salivary flow per minute was calculated based on the amount of saliva collected for 10 min.
2. **Salivary pH** was measured using the Eutech pH metre, PC 2700, with ion sensitive probes (Thermo Fisher Scientific, Waltham, Massachusetts, USA). Saliva consists of three buffer systems: bicarbonate, phosphate, and proteins, which help maintain a pH range within the oral cavity. The Eutech pH metre used for the measurement of pH was calibrated every day prior to the measurement. The electrode was dipped overnight in 0.1N hydrochloric acid. The pH metre was then calibrated with freshly prepared buffer solutions of pH 7 and pH 4. The electrode was placed in double distilled water following finer pH adjustments. The electrode was gently dried using filter paper prior to being dipped into the sample solution. The electrodes were gently washed into a stream of distilled water after each pH analysis.
3. **Salivary cortisol** was quantified using the Human Cortisol Elisa kit (Bioassay Technology Laboratory, Shanghai, China).
4. **Salivary anti-oxidant Capacity** was measured using the FRAP (Ferric Reducing Antioxidant Power) assay using the Elisa Kit (Bioassay Technology Laboratory).
5. **Salivary total protein** was assessed to help in the validation of the quantity of total protein in the secreted saliva. The BCA (bicinchoninic acid) assay was used for the analysis by the colorimetric method (Elabscience kit, USA).
6. **Viscosity of saliva** was analysed using Ostwald’s viscometer in comparison to water. This was determined by allowing the saliva to flow through the U-shaped tube with a circular cross section and measuring the rate of flow. Accordingly, in the case of the laminar flow of the fluids, the flow rate of the liquid can be denoted as the ratio of the pressure difference with respect to the viscous resistance. The value of the viscous resistance varies directly with respect to the viscosity of the fluid and the length of the tube. Ostwald’s viscometer method is a reliable, easy, and reproducible method to assess the viscosity of the fluid that works on the principle of Poiseuille’s law [29–32].
7. **Salivary microbial carriage** is vital for qualitative studies as it reflects the state of local microbiota and serves as a mirror for oral and systemic health status. Microbial carriage was evaluated by counting *Streptococcus mutans and Lactobacillus colonies* at a critical concentration of 105 CFU/mL. 1 ml of the collected whole saliva was transported to the laboratory in a bottle containing 1 ml of thioglycolate transport medium and processed on the same day. The sample was vortexed for 15 s with the Cyclomixer, CM 101. The culture media used were Mitis-Salivarius-Bacitracin agar for *S. mutans* and Rogosa agar for *Lactobacillus species* (Hi-Media Laboratories, Mumbai, India). 0.05 ml (50 µl) of saliva samples were transferred into petri dishes containing the culture medium using sterile micropipettes. Under aseptic conditions, the salivary samples were streaked onto the culture medium; to avoid any contamination of the samples, the inoculation procedure was done in a laminar air-flow chamber. The inoculated medium was then incubated at 37 degrees Celsius for 24–48 h. After the incubation period, the petri dishes were taken out of the incubator, and the colonies were counted with the colony counting grid (Medox-Bio Colony Counter). The colony count was expressed in CFU per millilitre (CFU/ml) of saliva.

### Study size and intervention

The sample size of this prospective study included a total of 135 participants. The significance level was fixed at 5% (α = 0.05), and the power of the study was calculated to be 95%. The effect size was estimated to be 0.32 (G Power 3.1). A well-informed written consent was obtained from all participants during recruitment.

Participants were asked to use the device continuously for 3 months, twice daily (morning and evening), for 10 min at a pre-programmed device frequency of 80 Hz. The frequency of 80 Hz was fixed following the use of the device during the pilot run, following assessment of salivary output, discomfort, pain, and/or other side effects. To assess the effectiveness of the device, Baseline and post-interventional salivary variables were analysed during the 0th and 12th weeks, respectively. However, analysis of variables was also measured in the 3rd and 6th weeks during the follow up.

### Device specification details

**Fig. 1:**
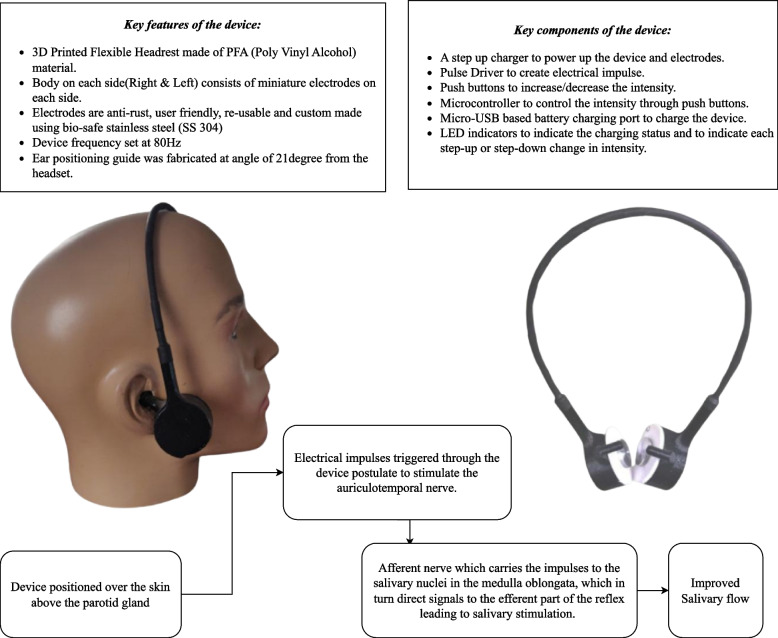
Illustrates the salient features of the device and the positioning of the device in a human model, also illustrating the principle of the device for salivary stimulation

A novel single unit wireless device with miniature electrodes (anti-rust, user-friendly, and reusable) was designed to generate electrical impulses over the parotid region to stimulate salivary output. As shown in Fig. 1, the device was a custom made 3D (three-dimensional) printed headrest designed with a body on each side (right and left) mounted with biocompatible electrodes on the medial aspect of the body on each side (right and left), which is positioned to rest over the skin over the parotid gland. Miniature electrodes were custom designed and developed using bio-safe stainless steel (SS 304). The device was custom designed to generate electrical impulses through the electrodes on the body (conductor) at a set frequency (80 Hz), which is hypothesised to stimulate the efferent nerves to improve the secretomotor drive and salivary output [19, 34].

### Statistical methods

*SPSS (IBM SPSS Statistics for Windows, Version 23.0, Armonk, NY: IBM Corp. Released 2015) was used for statistical analysis. Both parametric and nonparametric methods were applied for analysing the data.* Normality tests, Kolmogorov–Smirnov and Shapiro-Wilks test results revealed that some variables (pH, FRAP, and viscosity) followed normal distribution and some variables (cortisol, total protein, and microbial cultures) did not follow normal distribution.. The significance level was fixed at 5% (α = 0.05). The data was found to be statistically significant if the *p*-value was 0.05. Significance among different age-ranges was analysed by grouping the participants into 3 age-groups: 60 to 70 years, 70 to 80 years, and 80 years and above, respectively. Similarly, the significance of intervention in participants with systemic illness was also observed. Repeated measures ANOVA (general linear model) followed by the Greenhouse–Geisser-method for pairwise comparisons between time points were done. To compare the time points for cortisol, total protein, and microbial culture, repeated measures ANOVA (Friedman) and the Bonferroni adjusted Wilcoxon sign rank test were used.

## Results

We examined and recruited a total of 135 participants, out of whom 3 died during the study period due to old age, and 5 participants were excluded from the intervention due to illness. The final sample size available for statistical analysis was a total of 127. Participant demographics are given in Table 2. Salivary flow rate showed a significant increase to 0.35 ± 0.09 ml/min at the end of the 12th week from 0.15 ± 0.05 ml/min during the pre-intervention stage.

**Table 2:** Demographic data of the participants

Demographics	Study population (*n* = 127) n(%)
Age (years)	
60–70	63 (50)
70–80	42 (33)
80 and above	22 (17)
Gender	
Male	36 (28)
Female	91 (72)
Systemic Illness	
No illness	56 (44)
Diabetes	18 (14)
Hypertension	24 (18)
Diabetes & Hypertension	29 (23)

Gender-wise analysis revealed that significant changes were observed among both men and women at the end of 12 weeks. Salivary pH was found to be decreased in both men and women, but was found to be statistically significant among men; the salivary pH among men and women was to be 6.90 ± 0.79 and 6.85 ± 0.08 respectively. In both men and women, the salivary FRAP assay revealed a mild increase in the Total-Antioxidant capacity at the end of the 12th week (men: 31.71 0.94 μg/mL, Women- 29.89 0.63 μg/mL) but was not found to be statistically significant. Salivary viscosity at the end of the 12th week showed changes in both men (1.07 0.02cP) and women(1.03 0.01cP) but statistically significant only among women. Post-interventional salivary cortisol levels and microbial count [Lactobacillus sp. and Streptococcus mutans (S. mutans)] were found to be statistically significant among both men and women. (Refer Table 3).

**Table 3:** Gender wise comparison of the Salivary analytes Pre-interventional saliva (baseline) and Post-Interventional Saliva(12th week)

Gender	N	Parameter	Mean ± Sem	
			Pre- Interventional Saliva (Baseline)	Post-interventional Saliva (12^th^ week)
**Male**	**36**	pH	7.13 ± 0.15	6.90 ± 0.79 *
		FRAP (μg/mL)	30.27 ± 0.22	31.71 ± 0.94
		Viscosity (cP)	1.10 ± 0.03	1.07 ± 0.02
		Cortisol (μg/mL)	11.56 ± .54	8.48 ± 1.40 **
		Total Protein (μg/mL)	2.97 ± 0.24	3.30 ± 0.21
		Microbial culture "SM" (CFU/ml)	30.25 ± 0.54	25.67 ± 0.47*
		Microbial culture "L" (CFU/ml)	20.53 ± 0.35	16.36 ± 0.27*
**Female**	91	pH	6.93 ± 0.08	6.85 ± 0.08
		FRAP (μg/mL)	28.74 ± 0.30	29.89 ± 0.63
		Viscosity (cP)	1.10 ± 0.02	1.03 ± 0.01 **
		Cortisol (μg/mL)	10.13 ± 1.06	7.94 ± 0.83 **
		Total Protein (μg/mL)	3.21 ± 0.33	3.28 ± 0.34
		Microbial culture "SM" (CFU/ml)	20.63 ± 0.22	18.46 ± 0.19 *
		Microbial culture "L" (CFU/ml)	20.47 ± 0.21	15.89 ± 0.16 **

*FRAP* Ferric Reducing Antioxidant Power, *SM* Streptococcus mutans, *L* Lactobacillus count, *CFU* Colony Forming Unit, *cP* Unit of viscosity, *ml* millilitre, *μg* microgram

^*^p < 0.05 and **p < 0.001. Mean ± Sem (Standard Error of mean)

**Fig. 2:**
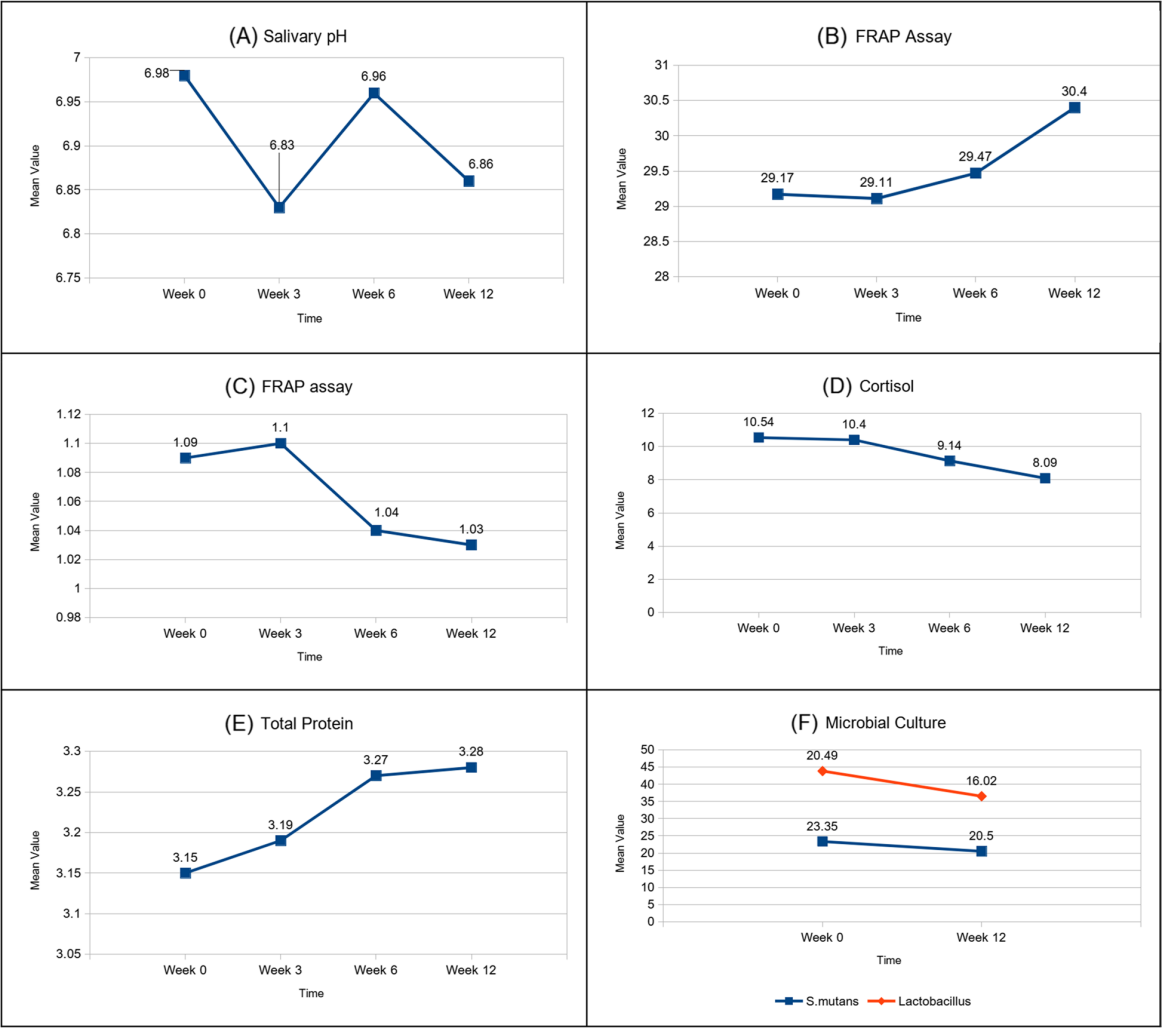
Shows the Pre & post-interventional change of salivary analytes. The linear graph shows change in the salivary analytes from Baseline to 12th week with significant difference from baseline. [FRAP—Ferric Reducing Antioxidant Power]

**Fig. 3:**
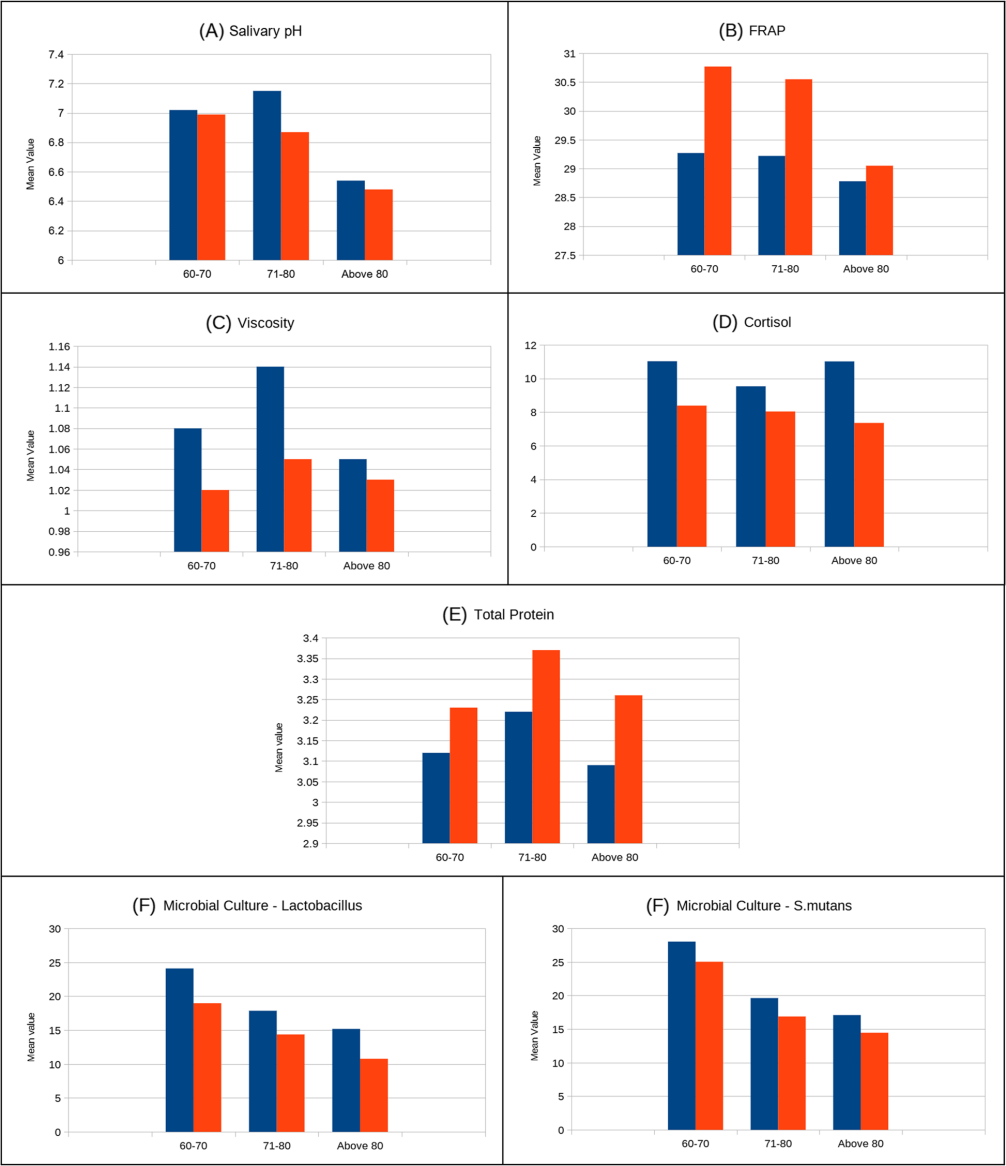
Shows the change of salivary analytes at different age groups (60—70,71—80, Above 80 years). [FRAP—Ferric Reducing Antioxidant Power]

Considering all participants together, the data revealed that there was a significant difference in the salivary analytes at the end of 12 weeks, except for total protein. Repeated measures for pairwise comparisons between time points were done and showed significance at *p* < 0.05 for salivary pH, the FRAP assay, and viscosity (Fig. 2A, B and C). Cortisol levels and microbial cultures showed a significant difference at *p* < 0.05 but total protein did not show any statistically significant change despite a minimal numerical change (Fig. 2D, E, and F). Age-wise analysis of the three age groups: 60–70, 71–81, and Above 80 years respectively. Overall, irrespective of age, there were changes in the salivary analytes at the end of the 12th week of the intervention (Fig. 3). Salivary pH showed a significant change in the 70-year-old group (Fig. 3A), and the FRAP (ferric-reducing antioxidant power) assay showed a significant change in the 60-year-old group (Fig. 3B). Viscosity showed significant change at both 60 and 70 years (Fig. 3C). Cortisol showed a significant change in all age groups (Fig. 4D). Total protein showed only numerical changes (3E). Microbial culture showed significant changes at 60 and 70 years in Lactobacillus (L) culture and at 60 years in Streptococcus mutans (SM) culture (Fig. 3F). Salivary changes among systemic illnesses such as diabetes and hypertension showed significance in pH, cortisol, and lactobacillus colony count. (Table 4).

**Table 4:** Comparison of Pre-interventional (Baseline) and Post-Interventional (12th week) Salivary analytes in common systemic illnesses. *p < 0.05 and **p < 0.001

Systemic illness	Parameter	Mean ± Sem	
		Pre-Interventional Saliva (Baseline)	Post-Interventional Saliva (12^th^ week)
**No illness**	pH	6.97 ± 0.12	6.88 ± 0.13
	FRAP (μg/mL)	28.80 ± 0.34	30.75 ± 0.78*
	Viscosity (cP)	1.10 ± 0.02	1.03 ± 0.02
	Cortisol (μg/mL)	10.45 ± 1.19	8.56 ± 0.94**
	Total Protein (μg/mL)	3.30 ± 0.19	3.47 ± 0.17
	Microbial culture "SM" (CFU/ml)	26.36 ± 0.40	22.70 ± 0.35*
	Microbial culture "L" (CFU/ml)	20.79 ± 0.26	16.54 ± 0.21**
**Diabetes**	pH	7.16 ± 0.16	6.78 ± 0.15*
	FRAP(μg/mL)	30.47 ± 0.24	32.01 ± 0.75
	Viscosity(cP)	1.122 ± 0.04	1.05 ± 0.03
	Cortisol(μg/mL)	9.48 ± 1.9	5.80 ± 1.30**
	Total Protein(μg/mL)	3.41 ± 0.5	3.24 ± 0.28
	Microbial culture "SM" (CFU/ml)	12.33 ± 0.37	11.83 ± 0.34
	Microbial culture "L" (CFU/ml)	13.56 ± 0.19	9.67 ± 0.27
**Hypertension**	pH	6.92 ± 0.10	6.79 ± 0.09
	FRAP(μg/mL)	29.29 ± 0.69	28.98 ± 1.02
	Viscosity(cP)	1.06 ± 0.03	1.01 ± 0.02
	Cortisol(μg/mL)	12.60 ± 1.04	10..22 ± 0.92*
	Total Protein(μg/mL)	2.65 ± 0.32	2.99 ± 0.22
	Microbial culture "SM" (CFU/ml)	28.88 ± 0.75	25.96 ± 0.69
	Microbial culture "L" (CFU/ml)	24.33 ± 0.49	19.88 ± 0.40*
**Diabetes & Hypertension**	pH	6.96 ± 0.15	6.96 ± 0.15
	FRAP (μg/mL)	29.00 ± 0.44	29.91 ± 0.14
	Viscosity (cP)	1.10 ± 0.03	1.06 ± 0.02
	Cortisol (μg/mL)	9.65 ± 0.79	6.86 ± 0.12**
	Total Protein (μg/mL)	3.09 ± 0.57	3.20 ± 0.59
	Microbial culture "SM" (CFU/ml)	19.83 ± 0.36	17.14 ± 0.31
	Microbial culture "L"(CFU/ml)	21.03 ± 0.39	15.79 ± 0.29

*FRAP* Ferric Reducing Antioxidant Power, *SM* Streptococcus mutans, *L* Lactobacillus count, *CFU* Colony Forming Unit, *cP* Unit of viscosity, *ml* Millilitre, *μg* Microgram

^*^p < 0.05 and **p < 0.001. Mean ± Sem (Standard Error of mean)

## Discussion

Ageing may cause several reversible and irreversible effects on the morphology and functions of several systems in the human body; likewise, the impact on the salivary secretory and ductal systems is reflected as Xerostomia (mouth dryness). Due to the known benefits of saliva in mastication, digestion, deglutition, taste, articulation, and speech, as well as its anti-inflammatory and immune-related properties, it is understood that mouth dryness largely impacts the quality of life among the geriatric population. Other systemic conditions and medications also contribute to the symptoms [33, 35].

In our systematic review of the non-pharmacological treatment modalities to treat xerostomia, evidence suggests that both intraoral and extraoral devices have the potential to increase salivary flow in patients with xerostomia. However, it was observed that extra-oral devices had better compliance among patients [21]. Our previous study was to design a novel single-unit wireless device and conduct a quantitative assessment of salivary stimulation. The primary objective of this study was to qualitatively assess the analytes of post-interventional unstimulated saliva in geriatric patients with xerostomia.

### Salivary flow

The mean baseline salivary flow rate was 0.15 ± 0.05 ml/min, while the mean salivary flow rate post-intervention at three months was 0.35 ± 0.09 ml/min (Table 5) [19]. Even though our study did not have a separate control group, baseline saliva was considered the control group in standardising the outcome.

**Table 5:** Comparison between Pre-Interventional Saliva (Baseline) and Post-Interventional Saliva (3rd, 6th,12th week)flow rate

Salivary flow rate	Mean ± SD (mL/min)	z-value	*P* value
**Baseline** Post-Interventional	0.15 ± 0.05 0.35 ± 0.09	9.888	< 0.001
Baseline 3rd week Post-Intervention 6th week Post-Intervention 12th week Post-Intervention	0.15 ± 0.05 0.16 ± 0.6 0.21 ± 0.9 0.35 ± 0.09	–	< 0.001

Several reported studies have proven an increase in salivary flow with the use of TENS. Dalbem et al. used TENS therapy for progressively increasing the salivary flow in patients with xerostomia and found evident change after 6 months of intervention [36]. A stable increase in salivary flow rate was shown in a study on the impact of the TENS device among haemodialysis patients. They also reported that the increased flow was effective for up to one week after the treatment [37]. Whole saliva was stimulated in the age range of 18–60 years in patients with xerostomia. According to their results, around 61% of the study group responded to TENS therapy [38]. Aggarwal et al. used a regular TENS device for stimulation (at 100 Hz) of the whole saliva in healthy adults and observed that there was an increase in the salivary flow rate. However, a few participants in their study group reported mild twitching of the facial musculature as a side effect [39]. Our study did not report any such side effects.

Resting saliva is usually primarily produced by the submandibular gland, and parotid secretions are mainly stimulated by masticatory actions or due to tastants. The role of TENS therapy in the stimulation of saliva along with masticatory function is unexplored. This can be evaluated by simultaneously assessing the masseter muscle activity and its effect on salivary output. In this study, transcutaneous stimulation with the precise placement of biocompatible electrodes over the skin of the parotid gland is hypothesised to stimulate the parotid gland via the postganglionic efferent auriculotemporal nerve fibres to produce the resultant salivary output. Several authors have already shown the potential of electrostimulation in both in-vivo and in-vitro cases of salivary gland regeneration, its effect on tissue engineering, and its improvement of salivary gland function [40–42].

### Salivary pH, total protein concentration & viscosity of saliva

Assessing salivary pH is a quick chairside test and an important marker for the quality of saliva. Literature suggests that the salivary flow rate is proportional to the salivary pH range, and maintaining the salivary pH within the range of 6.6 to 7.4 is considered to be ideal [43]. Our study recorded both pre-intervention and post-intervention unstimulated salivary pH at various time points to evaluate the changes in pH prospectively. At the end of the 12th week, it was observed that there were significant changes in salivary pH. The results of our study were similar to those of previous studies in which the salivary pH was evaluated in patients with dry mouth and found to be lower [44]. Further, it is observed that salivary flow rate and pH were directly proportional to the moisture of the oral mucosa [43, 45].

The moisture of the oral mucosa may also be related to other qualitative variables like the viscosity of saliva, which is again dependent on the salivary protein concentration, which has been further evaluated in this study. Total protein quantification in pre- and post-interventional saliva revealed only a minimal change in the total protein concentration between pre- and post-intervention. Lee et al. reported that the total salivary protein concentration measured by the Bicinchoninic acid (BCA) method in cases of hyposalivation had significantly higher values than in normosalivators [46, 47]. The study evaluated the relationship between a reduction in residual salivary volume and an increase in protein content and minor salivary gland secretions and suggested that there is a negative correlation between mucosal moisture and the protein concentration of residual saliva. The author suggested that this may be due to the fact that the function of the minor salivary glands was less damaged in patients with dry mouth [46, 47].

The viscosity of saliva may be attributed to various reasons, like side effects of medication, salivary duct stones, dehydration, radiation, dry mouth, etc. The consistency of saliva in xerostomia patients is stringy and thick as there is a decrease in the volume of saliva. Our device is solely aimed at stimulating the parotid gland, which is purely a serous salivary secretion. Hence, to evaluate the effective production of serous salivary output and its impact on the change in consistency of saliva, we chose to analyse the pre- and post-interventional viscosity of saliva. Our results showed an improvement in the viscous nature of salivary output in both males and females, which elicited clinically and proved to be statistically significant among female patients. In a previous study, the saliva secretion in completely edentulous patients was analysed, and the authors reported that changes in salivary viscosity were attributed to disease, medication, or changes due to dentures [48].

The consistency of saliva is directly proportional to the salivary flow and the protein concentration, which in turn are related to the cleansing and diluting properties of saliva. Hence, increased salivary flow suggests greater consistency, viscosity, buffering, and diluting capacity. Any change in the reduction of salivary flow would directly affect the oral environment. An increase in salivary flow or output corresponds to the salivary proteins and ions that are responsible for maintaining the pH, viscosity of saliva, and oral microbiota and thereby maintaining equilibrium. Sources for hydrogen ion concentration may be via secretion by the salivary glands in the form of organic and inorganic acids, food intake, or those produced by the microbiota. In our study, we were able to observe a significant improvement in salivary flow, which relates to the changes in the salivary buffering capacity (pH), protein concentration, and viscosity. It may be hypothesised that the use of TENS devices may directly play a role in improving the salivary flow and indirect role in the stimulation of qualitative variables like pH, protein concentration, and viscosity of saliva.

### Anti-oxidant capacity & salivary cortisol

The FRAP (Ferric Reducing Antioxidant Power) test measures the cumulative activity of all antioxidants present in bodily fluids and shows an overall integrated parameter as opposed to the basic measurable antioxidants [46–54]. Our data only showed a minimal increase in antioxidant levels post-intervention. Victor et al. reported that participants presented with low levels of total antioxidant levels and also suggested that antioxidant status in healthy older participants declined when compared with middle age [50].

Salivary cortisol levels are unaffected by salivary flow rate and are relatively resistant to degradation from enzymes or freeze–thaw cycles. The estimation of salivary cortisol reflects the stress levels among participants who have mouth dryness [51] and its assessment may help in assessing its impact on the quality of life of the patient. Venero et al. reported that salivary cortisol levels in older adults were increased due to mild cognitive impairment [52]. Studies of salivary cortisol levels in patients with dry mouth concluded that stress and anxiety alone may not be the main risk factors for the dry mouth; it could also be due to age-associated salivary gland hypofunction [53]. Whereas in our study, we chose to assess cortisol levels both pre- and post-intervention to observe if they were relieved of anxiety and stress-induced dry mouth. A significant increase in salivary flow with a corresponding increase in viscosity may clinically, translate to improved compliance of the individual as the oral wetness is improved or by relieving the oral dryness; this helps the patient have better speech articulation and chewing capability. It may be suggested that significant changes in salivary flow and clinical oral wetness may help to reduce stress among individuals as a result of changes in the hypothalamic–pituitary–adrenal (HPA) stress axis.

### Microbial colony count

Microbial carriage quantification is another vital analysis as it may reflect the status of oral health and general systemic health of an individual [54]. Hence, we assessed *Streptococcus mutans* and *lactobacillus* counts in all the participants, pre- and post-intervention. Our results showed a statistically significant decrease in the microbial colonies post-intervention, from which we could infer the patient’s immune or defensive response to the increased salivary output. The bacterial composition in the whole saliva of patients with severe hyposalivation was studied and inferred a varied comparable caries experience, and no single probe target was present with a significant difference in frequency [55]. Teare et al. observed in their study that patients with xerostomia were evaluated for more than 6 weeks, and candida albicans and staphylococcus aureus grew from the saliva of the patients [56]. In accordance with the available literature and our results, it is inferred that the bacterial profile of patients with xerostomia cannot be generalised; it differs to a great extent depending on pre-existing co-factors like untreated active caries lesions, periodontal disease, systemic illness, etc. Improved salivary buffering capacity, protein concentration, and antioxidant capacity as a result of Increased salivary flow indirectly supports the hypothesis of increased salivary defense response, thereby reducing the microbial colony count at the end of 12 weeks. However, the measure of microbial carriage is a good indicator of immune status in the absence of other cofactors. The role of other confounding factors (both local and systemic) should be critically assessed, as they may play a role in the quality of the secreted saliva.

Blom et al. suggested that acupuncture increases salivary secretion by increasing the blood flow near the salivary gland [57]. Although acupuncture could be an alternative non-pharmacological method for improving salivary flow, the use of a TENS device is easier to use and can be used by the patients themselves. Blom et al. in another study, reported that the use of acupuncture in patients with xerostomia resulted in an increased amount of salivary calcitonin related gene-related peptide, a protein known to be related to salivary flow and oral mucosal trophic effects [58].

The use of acupuncture and TENS devices is known to show both clinical and biological effects when used to treat dryness of the mouth [59, 60]. Acupuncture is suggested to induce salivary secretion by improving the salivary blood flow, followed by stimulation of the autonomic nervous system, thereby helping in the resultant improved salivary output. Meanwhile, as hypothesised earlier, other authors have also suggested that electrostimulation to one or more arms of the salivary reflex arch may help to stimulate an increased salivary secretory response [14, 60].

Overall, the use of this novel, cost-effective low-level transcutaneous electro neurostimulator has shown promising results, with an improved salivary flow rate and a relative improvement in the qualitative variables of the saliva. One of the limitations is that although we assessed the patients periodically (3rd and 6th week), only the baseline and 12th week are reported here as the study was designed to evaluate a long-term effect as it was observed that short term electrostimulation may only induce minimal changes to the salivary flow. Hence, the 12th week (3rd month) was set as a standard for determining the quantity of the output for the study. However, one major constraint in our research was the duration of observation; to obtain reliable and highly significant results long-term, follow-up for a minimum of one year in a larger population would be essential. It is also suggested to further stratify the patients based on the onset of xerostomia to see if less affected glands respond better. It is essential to observe if there is any relapse or threshold to the salivary output following long-term usage. Degenerative changes, oxidative damages due to ageing, medications, or pathologies should also be taken into consideration as co-factors in improving salivary flow and subsequent protein production. Our study establishes a predictable effect of our novel transcutaneous electro-neurostimulator device on the stimulation of salivary flow and functional measures, thereby improving the state of oral dryness.

## Conclusion

Based on the data available through a literature search, no study has done a qualitative analysis of the pre- and post-intervention saliva. Neurostimulation for salivary stimulation is relatively new and has yet to be validated as a protocol or treatment modality despite promising results; further large scale clinical trials evaluating the salivary flow and other qualitative parameters of saliva will elucidate the true potential of using neurostimulation for salivary stimulation. Our study with the novel device currently proves its efficacy to serve as a complementary therapy to improve patients' salivary flow and relevant qualitative parameters.

## Data Availability

The datasets used and/or analysed during the study are available from the corresponding author on reasonable request.
